# Diagnostic Usefulness of Real-Time Elastography for Liver Fibrosis in Chronic Viral Hepatitis B and C

**DOI:** 10.1155/2014/210407

**Published:** 2014-08-10

**Authors:** Young Woon Kim, Jung Hyun Kwon, Jeong Won Jang, Min Ju Kim, Byong Sun Oh, Kyu Won Chung, Eun Su Park, Soon Woo Nam

**Affiliations:** ^1^Department of Internal Medicine, Incheon St. Mary's Hospital, The Catholic University of Korea, Incheon 403-720, Republic of Korea; ^2^Department of Internal Medicine, Seoul St. Mary's Hospital, The Catholic University of Korea, Seoul 403-720, Republic of Korea; ^3^Department of Pathology, Incheon St. Mary's Hospital, The Catholic University of Korea, Incheon 403-720, Republic of Korea

## Abstract

The aim of this study was to investigate the diagnostic usefulness of real-time elastography (RTE) for liver fibrosis in chronic viral hepatitis B (CHB) and C (CHC). Fifty-one and thirty-two of the patients were diagnosed with CHB and CHC, respectively. Enrolled patients underwent liver biopsy and RTE. The FIB-4 index and aspartate transaminase-to-platelet ratio index (APRI) were also measured. The liver fibrosis index (LFI) by RTE increased significantly with the Knodell fibrosis stage: 3.14 ± 0.62 for F0, 3.28  ±  0.42 for F1, 3.43  ±  0.53 for F3, and 4.09  ±  1.03 for F4 (*P* = 0.000). LFI as well as APRI, FIB-4, platelet, albumin, and prothrombin time showed the difference in patients with advanced fibrosis (≥F3) and those with mild fibrosis (≤F1). In addition, RTE had better discrimination power between ≥F3 and F4 than between FIB-4 and APRI. In CHC patients, the area under receiver operating characteristic curves of RTE for advanced fibrosis was higher than that in CHB patients (0.795 versus 0.641). RTE is useful for the assessment of advanced fibrosis in patients with CHB and CHC and has better discrimination power than other serologic markers.

## 1. Introduction

Chronic liver diseases are a major cause of morbidity and mortality worldwide. In patients with a chronic liver disease, precise understanding of the hepatic fibrosis stage is important to estimate the prognosis. This is especially important in deciding an antiviral therapy for patients with chronic hepatitis B virus (HBV) infection, because those with advanced fibrosis need prompt treatment [1]. Liver biopsy is still the gold standard for the assessment of liver fibrosis [2]. However, it is an invasive procedure that has many procedure-related risks and is difficult to perform repeatedly to check progression of the fibrosis. Therefore, there has been increasing interest in noninvasive assessment of hepatic fibrosis in clinical practice using serum markers and scoring systems or noninvasive instruments and devices.

There have been many reports about fibroscan, which is a noninvasive device for the measurement of hepatic fibrosis [3–5]. Fibroscan can predict cirrhosis linked complications in chronic hepatitis B (CHB) patients [6] as well as recurrence of hepatocellular carcinoma after curative resection [7]. However, liver stiffness measurements using fibroscan can be difficult in obese patients or in those with narrow intercostal spaces, and it is impossible in patients with ascites [8]. Real-time elastography (RTE) is a new device for assessing tissue elasticity that can be sonography based method [9]. It is technically different from fibroscan, which measures the propagation speed of shear waves [10–12]. RTE captures 2D strain images induced by internal heartbeats, and the strain images show progressively increasing patchiness with increasing severity of fibrosis [9, 13]. Therefore, it can be possible to perform in obese patients or with ascites differently from transient elastography.

In a recent report, RTE is more accurate than transient elastography for measurement of significant fibrosis [10]. Also RTE is reported to be effective in patients with chronic hepatitis C (CHC) but not in patients with nonalcoholic fatty liver disease [14]. However, there are few reports on the diagnostic efficacy of RTE to measure hepatic fibrosis in patients with HBV [9, 13–15]. The objectives of this study were to assess hepatic stiffness using RTE in patients with CHB and CHC and to investigate its diagnostic usefulness for hepatic fibrosis.

## 2. Materials and Methods

### 2.1. Patients

Eighty-three consecutive patients, who performed liver biopsy for the staging of hepatic fibrosis at Incheon St. Mary's Hospital, the Catholic University of Korea, between 2011 and 2013, were enrolled. All patients were diagnosed with CHB and CHC and had not been previously treated with antiviral agents. Chronic viral hepatitis was diagnosed by the presence of HCV antibodies and HCV RNA, or hepatitis B surface antigen (HBsAg) in the serum for more than 6 months. In all patients, the liver fibrosis index (LFI) by RTE was measured simultaneously with liver biopsy. All procedures that followed were in accordance with the ethical standards of our institution (Catholic Medical Center Human Research Protection Program) and the ethics committee deemed that patient consent was not required as the samples used were retrospective.

### 2.2. The Measure of Liver Fibrosis Index by Real-Time Elastography

RTE was performed using a Hitachi Avius device (Hitachi Medical, Tokyo, Japan) and a linear probe (EUP-L52; central frequency, 5.5 MHz). The tissue elasticity distribution can be assessed by the strain and stress within the regions of interest (ROI). The linear probe was placed on the right lobe of the liver through an intercostal space, with the patient lying supine. A rectangular area which was free from large vessels, measuring 30 mm in length and 20 mm in breadth and 10 mm below the surface of liver, was chosen. The equipment automatically captured the internal distortion of the liver tissue by the beating of heart. In addition, to obtain good images, scanning was performed to avoid large vessels and attenuation by the lungs and ribs [16]. RTE provides a relative-strain image; thus, there should be no artifacts in the regions of interest of the strain image. All measurements of liver stiffness were performed by the same scanner to avoid interobserver bias. Numerical strain values for the pixels were converted into a color image within the rectangular area evaluated, ranging from 0 (red) at the minimal degree of hardness to 255 (blue) at the maximal degree of hardness, and a histogram was generated. Nine image features were extracted from each RTE image: the mean of relative strain value (MEAN), standard deviation of the relative strain value (SD), ratio of the blue area in the analyzed region (%AREA), complexity of the blue area (COMP), kurtosis of the strain histogram (KURT), skewness of the strain histogram (SKEW), entropy (ENT), inverse difference moment (IDM), and angular second moment (ASM). Multiple regression analyses were then performed with these nine image features to quantify the LFI according to the following formula:

(1) LFI=−0.009×MEAN−0.005×SD+0.023×%AREA+0.025×COMP+0.775×SKEW−0.281×KURT+2.083×ENT+3.042×IDM+39.979×ASM−5.542.

See [15, 17].

The mean LFI was determined from 10 images.

### 2.3. Assessment of Liver Histology

Ultrasound-guided percutaneous liver biopsies were performed, using a suction technique, with a needle 1.6 mm in diameter and 150 mm long. Specimens were fixed in formalin and embedded in paraffin. The liver biopsy specimens were stained with hematoxylin and eosin and Masson-trichome. Liver biopsies with fewer than five portal tracts (except for cirrhosis) were excluded from the histologic diagnosis. Pathologists who were blinded to all patient clinical data scored the fibrosis from F0 to F4 according to the Knodell Histological Activity Index (F0, no fibrosis; F1, fibrous portal expansion; F3, bridging fibrosis, i.e., portal-portal or portal-central linkage; F4, cirrhosis). There is no stage F2 in the Knodell Histological Activity Index [18]. Advanced fibrosis is defined as ≥F3 and minimal fibrosis is defined as ≤F1.

### 2.4. Serum Markers of Fibrosis

Complete blood cell counts and blood chemistry assays including aspartate aminotransaminase (AST), alanine aminotransaminase (ALT), alkaline phosphatase (ALP), albumin, *γ*-glutamyl transpeptidase (GGT), and total bilirubin were checked when the liver biopsies were performed. International normalized ratio of prothrombin time (PT INR) and Child-Pugh score were also measured. There have been several reports of APRI and the FIB-4 index as serum markers of hepatic fibrosis [19]. APRI and the FIB-4 index were calculated as follows [19, 20]:

(2) APRI=[(AST  of  the  sampleupper  limit  of  normal  range)×100]×(platelet  count  (109/L))−1,FIB-4=[(age×AST)(platelet×ALT  1/2)].

### 2.5. Statistical Analysis

Data were expressed as means ± SD or as medians and range. Significant differences were assessed using chi-square and Fisher's exact tests. The correlations between the LFI and the histologic fibrosis stage were assessed using Spearman's correlation coefficient. Differences were considered statistically significant at *P* < 0.050. Box plots were used to study the distribution of the LFI according to the patient's liver fibrosis. The diagnostic performances of the LFI, APRI, the FIB-4 index, and serum markers were assessed by receiver operating characteristic (ROC) curves. The areas under the ROC curves (AUCs) were calculated with 95% confidence intervals (CIs). Optimal cut-off values for each fibrosis stage were chosen by maximizing the sensitivity, specificity, and diagnostic accuracy. Factors predicting advanced fibrosis were identified using logistic regression analyses. Statistical analyses were performed using SPSS version 18.0 (SPSS Inc., Chicago, IL, USA).

## 3. Results

### 3.1. Baseline Characteristics of Patients

Of the 83 enrolled patients, 51 (61.4%) had HBV and 32 (38.5%) had HCV (Table 1). The histologic fibrosis stages were F0 in 14 patients (16.3%), F1 in 26 patients (31.3%), F3 in 26 patients (31.3%), and F4 in 17 patients (20.4%). Age, PT INR, and Child-Pugh score increased significantly with increasing severity of fibrosis (*P* = 0.000). Serum albumin levels and platelet counts decreased significantly with increasing severity of fibrosis (*P* = 0.000). There is no significant difference of AST and ALT according to the fibrosis stage.

### 3.2. Relationship between the LFI and Histologic Fibrosis Stage

**Figure 1:**
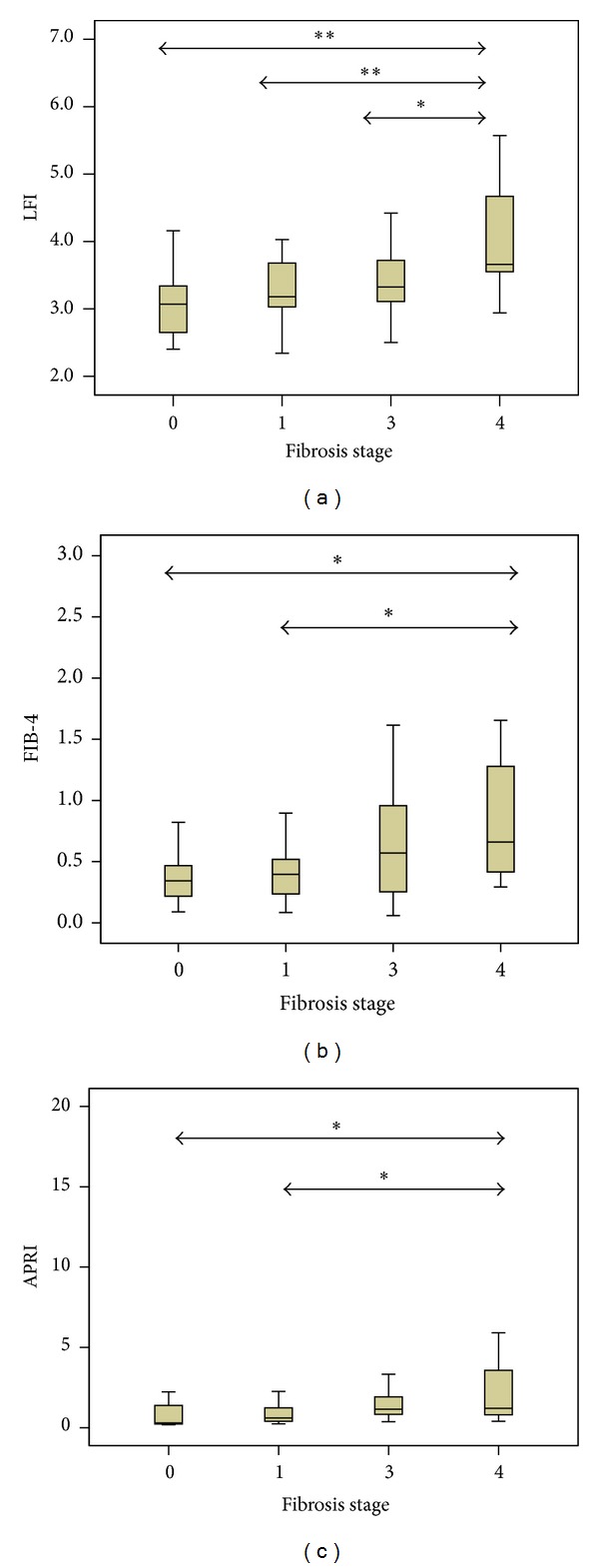
(a) Liver fibrosis index for each fibrosis stage. The increase of liver fibrosis index (LFI) with increasing fibrosis is shown and there is an evident separation between F0, F1, F3, and F4 groups. The lines through the middle of the boxes represent the means. The top and bottom of each box represents the 1st and 3rd quartiles. The length of the box represents the interquartile range within which 50% of the values were located (∗*P* < 0.05, ∗∗*P* < 0.001, comparing between each fibrosis stage). (b) FIB-4 index for each fibrosis stage. There were significant differences in F0 and F1 with F4 (∗*P* < 0.05, comparing between each fibrosis stage). (c) Aspartate aminotransferase/platelet ratio index (APRI) for each fibrosis stage (∗*P* < 0.05, comparing between each fibrosis stage).

The mean LFI by RTE significantly increased with the histologic fibrosis stage: 3.13 ± 0.62 for F0, 3.28 ± 0.43 for F1, 3.46 ± 0.52 for F3, and 4.09 ± 1.03 for F4 (*P* = 0.000) (Figure 1). The LFI was successfully obtained in all patients even with severe obesity and ascites because RTE had the advantage of being able to image liver stiffness in real time. There were significant differences of the LFI in stage F4, cirrhotic patients compared to the patients with the other histologic stages F0, F1, and F3 (Figure 1). There was a strong positive correlation between the histologic liver fibrosis stage and the LFI (Spearman's correlation coefficient = 0.39, *P* < 0.010). For predicting advanced fibrosis (stage ≥ F3) and cirrhosis (stage F4), the AUCs of the LFI obtained by RTE were 0.683 (95% CI 0.596~0.797) and 0.744 (95% CI 0.610~0.878), respectively (Table 2). The cut-off LFI value of >3.51 indicated a sensitivity of 82.4% and a specificity of 68.2% for predicting cirrhosis (stage F4).

### 3.3. Relationship between the LFI and Serologic Markers

**Figure 2:**
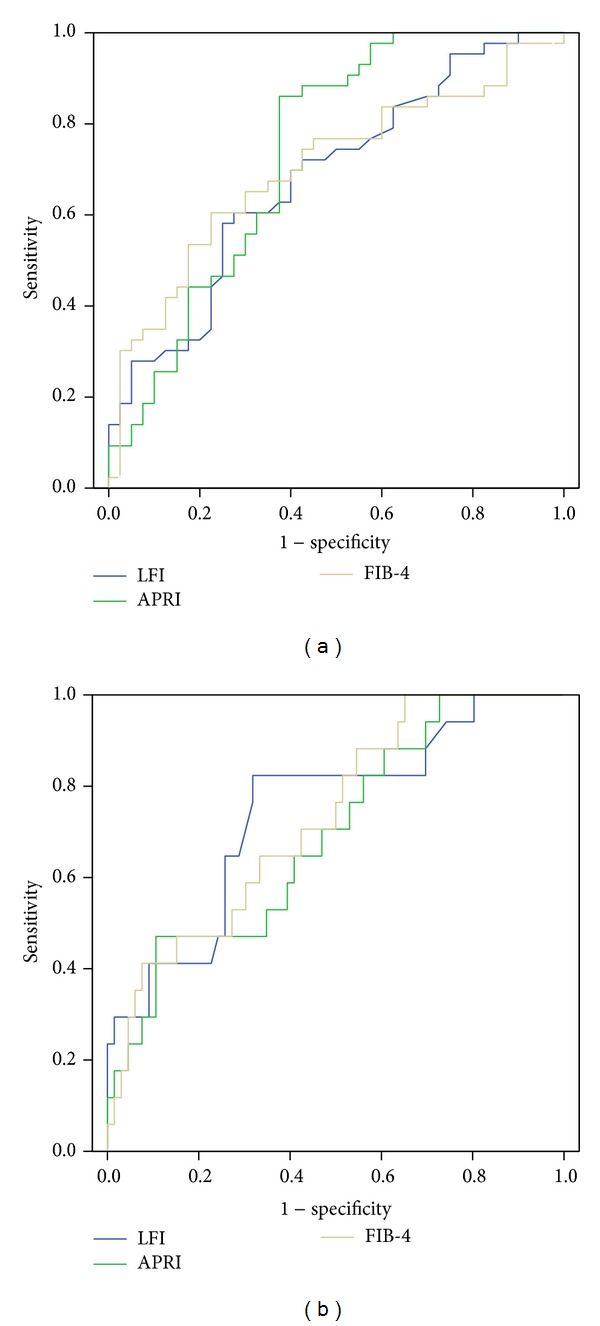
Receiver operating curves (ROC) curve of liver fibrosis index (LFI), aspartate transaminase platelet ratio index (APRI), the FIB-4 index for the prediction of advanced fibrosis (stage ≥ F3, (a)), and cirrhosis (stage F4, (b)).

APRI, FIB-4, and LFI in the patients with stage ≤ F1 were significantly lower than in those with stage ≥ F3 (*P* < 0.050; Figure 1). However, for discriminating stage F3 and F4, only LFI had a significant power (*P* < 0.050, Figure 1) for predicting the stage F4, and LFI showed higher AUC compared to APRI and the FIB-4 index (Table 2, Figure 2). In univariate analysis, platelet count, albumin level, Child-Pugh score, APRI, the FIB-4 index, and the LFI were significant factors for the diagnosis of cirrhosis. In multivariate analysis, only LFI proved to be the significant factor (odds ratio 3.840, 95% CI 1.306~11.295) (Table 3).

### 3.4. Combined LFI and Serologic Marker for the Diagnosis of Liver Fibrosis

We examined the diagnostic performance of various combination formulas using LFI and APRI and FIB-4. Combination formula of LFI multiplied by APRI showed the best AUROC for the prediction of stage ≥ F3 (0.754, 95% CI 0.648~0.861) compared to LFI, APRI, FIB-4, and LFI ∗ FIB-4. Also for discriminating stage F4, combination formula of multiplying LFI by FIB-4 showed the best AUROC (0.762, 95% CI 0.643~0.881). With regard to the prediction of advanced fibrosis, the combination formula of LFI and serologic parameters showed better AUROC than LFI, APRI, or FIB-4 alone.

### 3.5. Comparison of the LFI between CHB and CHC

In CHB patients, the predicting power for advanced fibrosis (stage ≥ F3) of RTE was lower than that in CHC patients. The AUCs of the LFI for predicting advanced fibrosis were 0.641 (95% CI 0.483~0.798) in CHB and 0.795 (95% CI 0.604~0.900) in CHC. However, there are no difference of APRI or FIB-4 index in the same fibrosis stage between CHB and CHC patients, although CHB patients showed higher mean ALT levels (*P* = 0.035) than the CHC patients.

## 4. Discussion 

This study suggests that RTE is useful diagnostic equipment for hepatic fibrosis in CHB and CHC patients. RTE shows a promise for use in patients for whom the application of Fibroscan may be limited. RTE can also discriminate between advanced fibrosis (F3) and cirrhosis (F4) more effectively than other serologic markers. Therefore, LFI by RTE was especially valuable for diagnosis of advanced hepatitis and early cirrhosis that are concerns in clinical practice.

In the present study, the LFI had a strong positive correlation with the stage of histological fibrosis as determined by the Knodell index. Liver biopsy has been the gold standard method for diagnosing liver fibrosis although it has shown significant intra- and interobserver variability and sampling errors [21–23]. In recent years, there have been many reports of assessment for hepatic stiffness without liver biopsy. Among them, fibroscan has been most frequently used in clinical practice [3–5]. However, fibroscan had some limitations in special patients [8]. Furthermore, examination with fibroscan often requires the use of ultrasonography to find the good window because there is no B-mode. The average failure rate is 3.1% and highly depends on body mass index. Measurements are unreliable up to 15.8% of cases [24]. By contrast, RTE displays in real time the relative strain of the tissue by measuring its displacement and it can easily find the most appropriate region and capture the value. In the present study, all patients could check the LFI by RTE, easily. The most recent report that RTE is more accurate than fibroscan for assessing hepatic fibrosis support RTE's feasibility and effectiveness [10]. In present study, RTE can discriminate between advanced fibrosis (F3) and cirrhosis (F4) as well as significant fibrosis (≥F3). In patients with chronic viral hepatitis, the diagnosis of those with advanced fibrosis is of special issue because it is an important indication for antiviral treatment [1]. Therefore, RTE with a high diagnostic accuracy for the determination of advanced fibrosis is of great therapeutic value comparable to the APRI and FIB-4 index. Combining serologic marker with LFI (multiplying LFI by APRI, LFI by FIB-4) improved the diagnostic performance.

The present study suggests that the LFI in patients with CHC was more predictable than in patients with CHB. A few recent published reports about RTE reviewed mostly the patients with CHC [9, 25]. Classically, the patients with CHB have the dynamic course of viral titers and ALT levels comparable to those with CHC. In report for fibroscan, the patients with high ALT showed the trend of higher score than those with normal ALT levels [26, 27]. However, in the present study, there are no difference of LFI among the same fibrosis stage between CHB and CHC patients although CHB patients show higher mean ALT levels than the CHC patients. Further studies are needed to fully explore the difference of diagnostic power of RTE in CHB and CHC patients.

The limitations of this study were that the degree of fatty infiltration was not reflected. One study reported that the LFI calculated using RTE was less useful for the evaluation of liver fibrosis in patients with nonalcoholic fatty liver disease than in CHC [14]. However, all evaluated patients in present study had been diagnosed with chronic viral hepatitis and their mean BMI was less than 24 (kg/m^2^), which suggested that the presence of nonalcoholic fatty liver disease was relatively small compared to the western patients.

## 5. Conclusion 

In conclusion, RTE can be easily used in patients for whom the application of fibroscan is difficult and has better discrimination power for advanced fibrsosis and cirrhosis than other serologic markers. RTE is promising new sonography-based noninvasive equipment for the assessment of hepatic fibrosis in patients with CHB and CHC.

## Figures and Tables

**Table 1:** Baseline characteristics of patients according to the histologic fibrosis stage.

Fibrosis stage	F0 (*n* = 14)	F1 (*n* = 26)	F3 (*n* = 26)	F4 (*n* = 17)	Total (*n* = 83)	*P* value
HBV/HCV	5/9	13/13	17/9	16/1	51/32	
Sex, male/female	8/6	16/10	18/8	12/5	54/29	
Age, years	38 ± 11	47 ± 6	47 ± 10	48 ± 12	45 ± 10	0.031
BMI, kg/m^2^	24.2 ± 2.5	24.1 ± 2.9	23.6 ± 3.5	24.0 ± 2.6	23.9 ± 2.9	0.912
AST, IU/L	59 ± 75	85 ± 85	82 ± 54	156 ± 207	94 ± 116	0.086
ALT, IU/L	101 ± 161	137 ± 177	134 ± 129	143 ± 182	131 ± 159	0.895
GGT, IU/L	73 ± 106	88 ± 111	62 ± 40	81 ± 82	76 ± 86	0.746
ALP, IU/L	89 ± 52	73 ± 24	85 ± 28	85 ± 33	82 ± 33	0.454
TB, mg/dL	1.3 ± 1.8	0.8 ± 0.2	1.0 ± 0.7	2.0 ± 3.2	1.2 ± 1.7	0.141
Albumin, g/dL	4.1 ± 0.4	4.0 ± 0.3	3.8 ± 0.4	3.4 ± 0.6	3.8 ± 0.5	0.000
Platelet, 10^3^/mm^3^	218 ± 63	207 ± 51	149 ± 52	128 ± 39	174 ± 62	0.000
PT INR	1.04 ± 0.09	1.06 ± 0.06	1.15 ± 0.12	1.22 ± 0.16	12.90 ± 1.60	0.000
Median CP score (Range)	5.36 (5~6)	5.21 (5~6)	5.36 (5~7)	5.83 (5~8)	5.39 (5~8)	0.028

HBV, hepatitis b virus; HCV, hepatitis c virus; BMI, body mass index; ALT, alanine aminotransferase; AST, aspartate aminotransferase; ALP, alkaline phosphatase; TB, total bilirubin; GGT, *γ*-glutamyl transpeptidase; PT INR, prothrombin time international normalized ratio; CP, Child-Pugh.

**Table 2:** Areas under receiver operating characteristic (ROC) curves of liver fibrosis index and several serological makers for predicting liver fibrosis stage.

	F ≥ F3			F = F4		
	AUC	95% CI	*P* value	AUC	95% CI	*P* value
LFI	0.683	0.596~0.797	0.004	0.744	0.610~0.878	0.002
APRI	0.737	0.627~0.847	0.000	0.694	0.557~0.832	0.014
FIB-4 index	0.702	0.589~0.816	0.002	0.729	0.602~0.856	0.004

AUCs, areas under receiver operating characteristic (ROC) curves; CI, confidence interval; LFI, liver fibrosis index; APRI, aspartate aminotransferase/platelet ratio index.

**Table 3:** Univariate and multivariate logistic regression of predicting F4 stage.

Univariate				Multivariate		
	*P* value	Odd ratio	95% CI	*P* value	Odd ratio	95% CI
Platelet	0.002	0.978	0.964~0.992	0.292	0.991	0.974~1.008
Albumin	0.001	0.097	0.024~0.384	0.664	0.653	0.095~4.472
PT INR	0.003	1713.397	12.145~241715.183	0.103	189.525	0.346~103706.928
LFI	0.001	4.6	1.799~11.763	0.015	3.840	1.306~11.295
ALT	0.73	1.001	0.997~1.004			
Total bilirubin	0.087	1.327	0.959~1.835			
CP score	0.008	1.919	1.183~3.113			
APRI	0.019	1.443	1.063~1.960	0.156	1.214	0.929~1.586
FIB-4 index	0.004	5.684	1.715~18.839			

CI, confidence interval; PT INR, prothrombin time international normalized ratio; LFI, liver fibrosis index; ALT, alanine aminotransferase; CP, Child-Pugh; APRI, aspartate aminotransferase/platelet ratio index.
